# Ammonium nitrate regulated the color characteristic changes of pigments in *Monascus purpureus* M9

**DOI:** 10.1186/s13568-020-01165-6

**Published:** 2021-01-04

**Authors:** Di Chen, Yurong Wang, Mianhua Chen, Pei Fan, Guiling Li, Changlu Wang

**Affiliations:** 1 College of Biological Engineering, Henan University of Technology, No. 100, Lianhua Street, High-tech Industrial Development Area, Zhengzhou, 450001 People’s Republic of China; 2 Key Laboratory of Food Nutrition and Safety, Ministry of Education, College of Food Engineering and Biotechnology, Tianjin University of Science and Technology, No. 29, 13th Avenue, TEDA, Tianjin, 300457 People’s Republic of China

**Keywords:** *Monascus* pigments, Color characteristics, Pigments conversion, Gene expression, Ammonium nitrate

## Abstract

*Monascus* pigments (MPs) with different color characteristics, produced by submerged fermentation of *Monascus purpureus* M9, have potential application in food industry. In the present study, the effects and regulatory mechanisms of ammonium nitrate (AN) on the color characteristics of MPs were investigated. The concentration of intracellular pigments was significantly decreased when subjected to AN. The hue and lightness value indicated AN altered the total pigments appearance from original red to orange. The HPLC analysis for six major components of MPs showed that the production of rubropunctatin or monascorubrin, was significantly reduced to the undetectable level, whereas the yields of monascin, ankaflavin, rubropunctamine and monascorubramine, were apparently increased with AN supplement. To be noted, via real-time quantitative PCR strategy, the expression level of *mppG*, closely relative to orange pigments biosynthesis, was significantly down-regulated. However, the expression of *mppE*, involved in yellow pigments pathway, was up-regulated. Moreover, the broth pH value was dropped to 2.5–3.5 in the fermentation process resulted from AN treatment, along with the increased extracellular polysaccharide biosynthesis. Taken together, the change of MPs categories and amounts by AN might be the driving force for the color characteristics variation in *M. purpureus* M9. The present study provided useful data for producing MPs with different compositions and modified color characteristics.

## Introduction

Color pigments are widely used in food industry to restore color lost in processing or to improve the appearance of a product. The current trend is to replace artificial colorants with natural equivalents. The commonly used natural pigments include red pigments (cochineal, monascus) (Calvo and Salvador 2002), yellow (curcumin) (Upadhyaya et al. 2015), and green (chlorophyll) (Luo et al. 2019), in which the microorganisms-produced pigments possess a series of clear-cut advantages. For instance, they have good qualities for harvest, and are available for large-scale production, as well as are not subject to the vagaries of nature (Sen et al. 2019). *Monascus* pigments (MPs), the important secondary metabolites of *Monascus*, have been used as food colorants for thousands of years in East Asian (Chen et al. 2015; Feng et al. 2012). The annual production of MPs is estimated to be nearly 20,000 tons in China, and the market demand nationwide for MPs increases progressively with years (the annual growth rate is 5–10%) (Yang et al. 2015). Researches indicate MPs could exhibit several biological activities, such as anti-inflammation (Hsu et al. 2012), anti-cancer (Su et al. 2005), anti-microbe (Kim et al. 2006), and anti-obesity (Kim et al. 2007). Therefore, more than food colorants, MPs have potentials to the pharmaceutical, cosmetics, dyeing cotton and leather industries (Lin et al. 2017; Velmurugan et al. 2009). Currently, the major MPs products appear to be red or deep red, which in turn limits their applications. The rapid development of food industry, however, requires various color characteristics of pigments.

MPs are a complex mixture of compounds with a common azaphilone skeleton. They are mainly composed of yellow, orange and red pigments. Up to date, more than 90 MP members have been identified, which include 44 yellow, 8 orange and 42 red pigments based on recent reports (Chen et al. 2017; Patakova 2013). Among those the top six well-known pigments were 2 yellow (monascin and ankaflavin), 2 orange (rubropunctatin and monascorubrin), and 2 red (rubropunctamine and monascorubramine) (Jůzlová et al. 1996). Thus to find simple approaches to produce MPs with diversiform color characteristics will be greatly beneficial to the amelioration of their economic value.

It has been consistently shown that selective nitrogen sources largely influence the composition of MPs. Some studies showed that the red MPs production is significantly suppressed under nitrogen limitation (Lin et al. 2007). To be noted, red or orange pigments can be formed when yeast extract/nitrate or ammonium salt is supplemented, respectively (Carels and Shepherd 1977). Also others suggested that ammonium sulfate contributes to the formation of red pigments with a small amount of yellow ones. In contrast, peptone promotes the biogenesis of yellow pigments with a few red counterparts (Chen and Johns 1993; Shi et al. 2015). In our previous study, we found ammonium nitrate (AN) greatly inhibits the biosynthesis of citrinin, the nephrotoxic and hepatotoxic mycotoxin produced along with MPs. Interestingly, the fermentation broth are demonstrated the changed colors when AN is added (Chen et al. 2016). However, the underlying mechanisms for AN regulating color transformation are still unclear, and the accurate detection for individual pigment concentration is rarely reported.

Hence, to fully elucidate the role of AN in MPs biosynthesis, the color characteristics and composition of MPs were systematically analyzed by spectrophotometry, colorimetry and HPLC strategies in this study, followed by detection for cell growth and pH of fermentation broth, as well as exoploysaccharide excretion. The primary molecular mechanism of pigment biosynthesis, reflected by the expression of several key genes, was also compared by RT-qPCR in transcriptional level. Our data, for the first time, disclose the regulatory mechanisms of AN in the color characteristic changes of MPs, which are useful to produce multiple MPs in different forms and usages.

## Materials and methods

### Microorganism and culture conditions

*Monascus purpureus* M9 (strain NO. CGMCC 3.19586) was maintained on malt extract agar (MA) slants with a sugar content of 10°Bx at 4 °C. Strain M9 was cultivated on MA slant at 28 °C for 7 days. After that the slant was washed with 3 mL distilled water, followed by being transferred to the culture medium (glucose, 60 g/L; peptone, 20 g/L; KH_2_PO_4_, 10 g/L, NaNO_3_, 10 g/L, and MgSO_4_, 5 g/L; pH 4.5). The inoculum was incubated in 250 mL flask containing 100 mL of the culture medium for 36 h in a rotary HZQ-C shaker (HDL, Harbin, China) at 180 rpm and 28 °C. Spore suspension was obtained by filtering the aforementioned inoculum with 8-layer sterile gauze. The concentration of spore suspension was adjusted to 10^6^ spores/mL.

For pigment fermentation, 3 mL of spore suspension was inoculated into 50 mL of rice medium in 250 mL flask. Cultures were incubated at 30 °C and 180 rpm for 7 days. The fermentation culture medium was composed of rice powder, 50 g/L; KH_2_PO_4_, 1.5 g/L; NaNO_3_, 3 g/L and MgSO_4_∙7H_2_O, 1 g/L. The added NH_4_NO_3_ was at the concentration of 10 g/L, which was optimized in previous study (Chen et al. 2016).

### Measurement of biomass and pH

Biomass from fermentation broth was determined by dry mycelium weight (DMW). The mycelium were filtered from the culture broth, and then washed 3 times with distilled water. Dried at 60 °C in the oven to a constant weight, the dry mycelium was collected. The pH value of fermentation broth was determined by EF28 pH meter (Mettler-Toledo, Zurich, Switzerland).

### Preparation of MPs

Dry mycelium was ground thoroughly, then the powder of 0.5 g was transferred into a 10 mL centrifuge tube, with the addition of 75% ethanol of 3 mL. Pigments were extracted via incubating in an ultrasonic bath for 30 min and then centrifuged at 2862×*g* for 10 min. The experiment was in triplicate. All supernatant was merged and used to pigments detection.

### UV–VIS spectrophotometric analysis of MPs

The concentration of pigments was determined by a Cary-3500 UV/VIS spectrophotometer (Agilent, California, USA) in a range from 380 to 600 nm at 1-nm intervals. The absorbance units (AU) at specific wavelengths (410, 470 and 510 nm) were used as an index of the intracellular yellow, orange, and red pigments concentration. One unit of optical density corresponded to 1 U of color value:

$${\text{Color value }}\left( {{\text{U}}/{\text{g biomass}}} \right) = \, ({\text{A }} \times {\text{ dilution factor}} \times {\text{V}})/{\text{m}}$$

where A is the absorbance of the pigment extract at specific wavelengths, V is the total volume (10 mL) of pigments extract, and m is the dry cell weight (0.5 g) of mycelia used for pigment extraction.

### Colorimetric analysis of MPs

The color characteristics of pigments were analyzed by a CR-300 colorimeter (Minolta, Osaka, Japan) using pigments extract, of which the absorbance was adjusted to the range of 1.0–2.0. The CIELAB color space parameters reflect color features. L* indicates lightness from 0 (black) to 100 (white). Positives and negatives in a* represent red and green, respectively, whereas positives and negatives in b* represent yellow and blue, respectively; Cab* is the chroma where Cab* = [(a*)^2^ + (b*)^2^]^1/2^ and represents the saturation of color: chroma values closes to 0 indicate duller or grayer colors, higher values indicate more vivid colors; the hue angle (Hab), where Hab = tan^−1^(b*/a*), is a numerical value that represents the hue: hue angles of 0, 90, 180 and 270 represent red, yellow, green and blue, respectively (Dubois et al. 1956; Jung et al. 2003).

### HPLC analysis of MPs

The quantitative analysis of the six main MPs was performed by 1200 HPLC system (Agilent, California, USA) coupled with a diode array detector. Pigments were separated by a reverse-phase column (XDB C18, 150 mm × 4.6 mm, 5 μm, Agilent, California, USA) with a flow rate of 1.0 mL/min. The mobile phase was solvent A (0.1% formic acid in water) and solvent B (acetonitrile). A gradient elution was performed as follows: solvent B was maintained at 60% for 12 min, 60 to 90% for 13 min, 90% for 2 min, 60 to 90% for 2 min. The detection wavelength was 410 nm. Sample in the volume of 20 µL was used for each experiment.

### Real-time quantitative PCR analysis of pigments biosynthetic genes

Fresh mycelia were collected from fermentation broth and were kept in liquid nitrogen for RNA extraction. Total RNA was extracted from mycelia using the Plant RNA Kit (Omega, USA). First-strand cDNA was synthesized using the PrimeScript 1st Strand cDNA Synthesis Kit (TaKaRa, Japan). All primers used in this study are listed in Additional file 1: Table S1. RT-qPCR was performed using the Stratagen Mx3000P (Agilent, California, USA) with the following cycling program: hold at 95 °C for 30 s, followed by a three-step PCR (42 cycles of denaturation at 95 °C for 5 s, annealing at 60 °C for 30 s, and extension at 72 °C for 30 s) and dissociation curve analysis (at 95 °C for 15 s, annealing at 60 °C for 30 s, then collecting the dissociation curve from 60 °C to 95 °C, finally at 95 °C for 15 s). The relative levels of target mRNAs were determined using the 2^−ΔΔCt^ method and were normalized to the β-actin mRNA signals in each sample.

### Determination of extracellular polysaccharide

Fermentation broth of 10 mL was filtered and all filtrates were collected. The crude polysaccharide was precipitated with the addition of four volumes of 95% ethanol for 12 h. The precipitated polysaccharide was collected by centrifugation at 2683×*g* for 10 min. After that precipitates were redissolved in 10 mL distilled water with 3% peroxide for 3 h at 60 °C to remove pigments. Total polysaccharide in the culture medium was determined by Spark 10 M ELIASA (Tecan, Männedorf, Switzerland) according to phenol–sulphuric acid assay (Cuesta et al. 2003).

### Statistical analysis

Each experiment was repeated at least 3 times. Numerical data are presented as mean ± SD. The differences between groups were analyzed using one-way ANOVA. All statistical analysis was performed by using SPSS 17.0 software. P value < 0.05 and < 0.01 were considered statistically significant.

## Results

### AN decreased both biomass and pH of fermentation broth

**Fig. 1:**
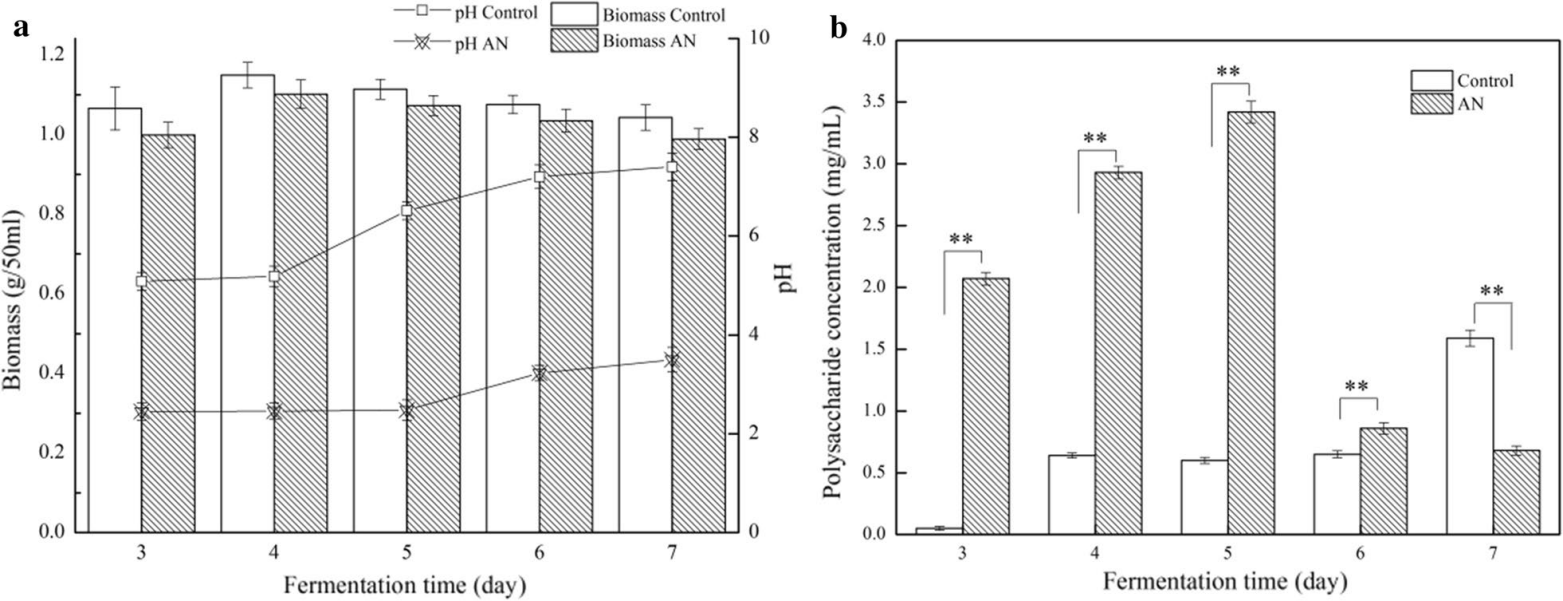
Effects of ammonium nitrate (AN) on biomass, pH of fermentation broth (**a**) and extracellular polysaccharide production (**b**). Biomass was estimated by determining dry mycelium weight. The extracellular polysaccharide was measured by phenol–sulphuric acid assay. The data were represented as the mean ± SD (n = 3). *P < 0.05, **P < 0.01 was compared with control

The extracted pigments from mycelium in the process of fermentation without AN was served as the control sample. DMW was increased at initial stage of fermentation (1–4 days), and then decreased in the late (5–7 days). The maximum amount of mycelium was obtained on the 4th day of fermentation. Biomass with AN supplement was slightly lower than the control sample on each day, but not to the significant level (Fig. 1a). However, the pH of fermentation broth with AN supplement was significantly reduced at each day point compared to that of the control. To be exact, the initial pH value was 5.0, and the final pH value reached to 7.0 for the control sample, whereas the pH values of fermentation broth treated by AN were changed from 2.5 to 3.5 (Fig. 1a). The pH decreased remarkably when exposed to AN, the phenomenon might be caused by the fact that AN is strong acid-weak base, tending to react with OH^−^ to form NH_3_ · H_2_O (weak alkali), which resulted in increasing the concentration of dissociative H^+^ in liquid medium (Peters and Smuła-Ostaszewska 2012).

### AN reduced the concentration of MPs

**Fig. 2:**
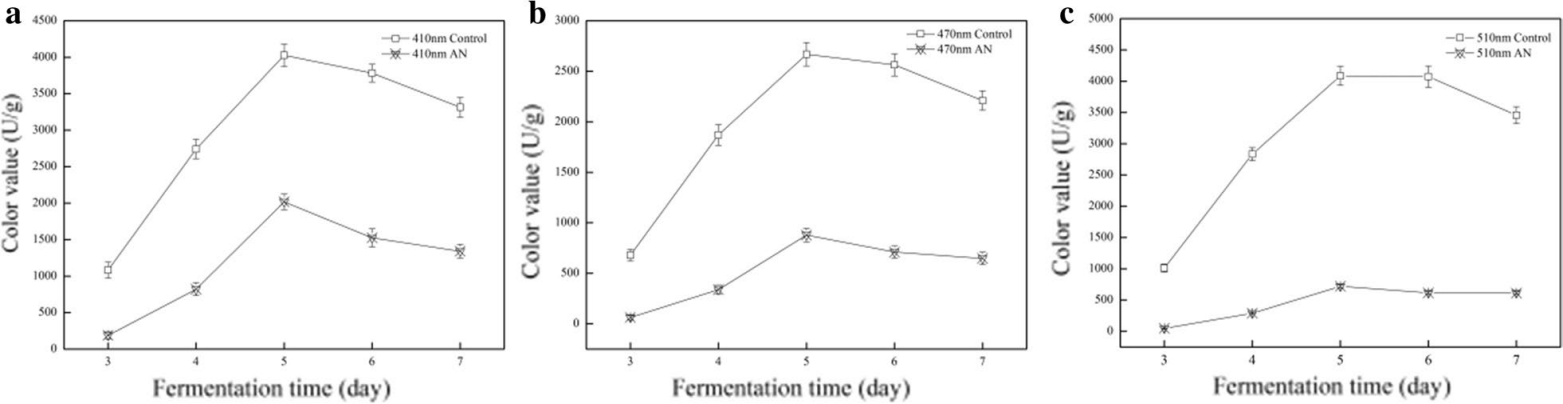
Effects of AN on the concentration of MPs. The concentration of yellow, orange, and red pigments was assessed by absorbance at 410 nm (**a**), 470 nm (**b**) and 510 nm (**c**) from the 3rd to 7th day of fermentation, respectively. The color values were expressed as OD units per gram of dry mycelia. The data were represented as the mean ± SD (n = 3)

It was widely accepted that the absorbance was measured at 410 nm for yellow pigments, 470 nm for orange pigments and 510 nm for red pigments (Chen et al. 2015). In this work the intracellular pigments were investigated from the 3rd to 7th days of fermentation, due to the ratios of intracellular to extracellular pigments were approximately 200:1. The results showed that yellow, orange and red MPs production presented a maximum amounts on the 5th day, reaching to 4025, 2688 and 4086 U/g in the control sample, respectively (Fig. 2). Whereas the concentrations of these MPs significantly decreased with AN supplementation, especially orange and red pigments, which reduced by 67.1% and 82.3% at most compared to the control samples, respectively (Fig. 2b, c). The data indicated AN resulted in the decrease of MPs concentrations.

### AN altered the color characteristics of MPs

The lightness value of MPs in the control sample were in a range from 32 to 5 as fermentation proceeded, and the hue angles decreased from 40 to 25, corresponding to the color ranging from orangish red to deep red. However, the higher values compared to the control that the hue angles of 80–40 and lightness value of 88–36 were obtained with AN supplement, of which the corresponding color were from light orange to orangish red (Table 1). These data were in agreement with the color change of fermentation broth (Fig. 4c). In deed, AN led to the visual color of MPs changing from red to orange.

**Table 1:** The color characteristics of MPs with AN treatment and the control samples

Group	Fermentation time (days)	CIELAB color system					Visual color^a^
		L*	a*	b*	C_ab_^b^	H_ab_^c^	
Control	3	32.67	60.70	50.94	79.24	40.00	Orangish red
	4	19.11	50.86	32.94	60.60	32.93	Orangish red
	5	5.53	4.66	3.96	6.11	40.35	Orangish red
	6	5.96	4.51	2.40	5.11	28.08	Deep red
	7	5.69	4.92	2.33	5.44	25.37	Deep red
NH_4_NO_3_	3	88.79	4.75	27.70	28.10	80.26	Light orange
	4	54.61	47.25	66.31	81.42	54.46	Middle orange
	5	42.36	57.16	70.24	90.56	50.86	Middle orange
	6	44.72	57.71	69.34	90.21	50.23	Middle orange
	7	36.51	61.71	61.41	87.06	44.86	Orangish red

^a^The colors were distinguished by visual inspection

^b^C_ab_ = [(a*)^2^ + (b*)^2^]^1/2^

^c^H_ab_ = tan^−1^(b*/a*)

### AN transformed the six classical MPs production by HPLC

**Fig. 3:**
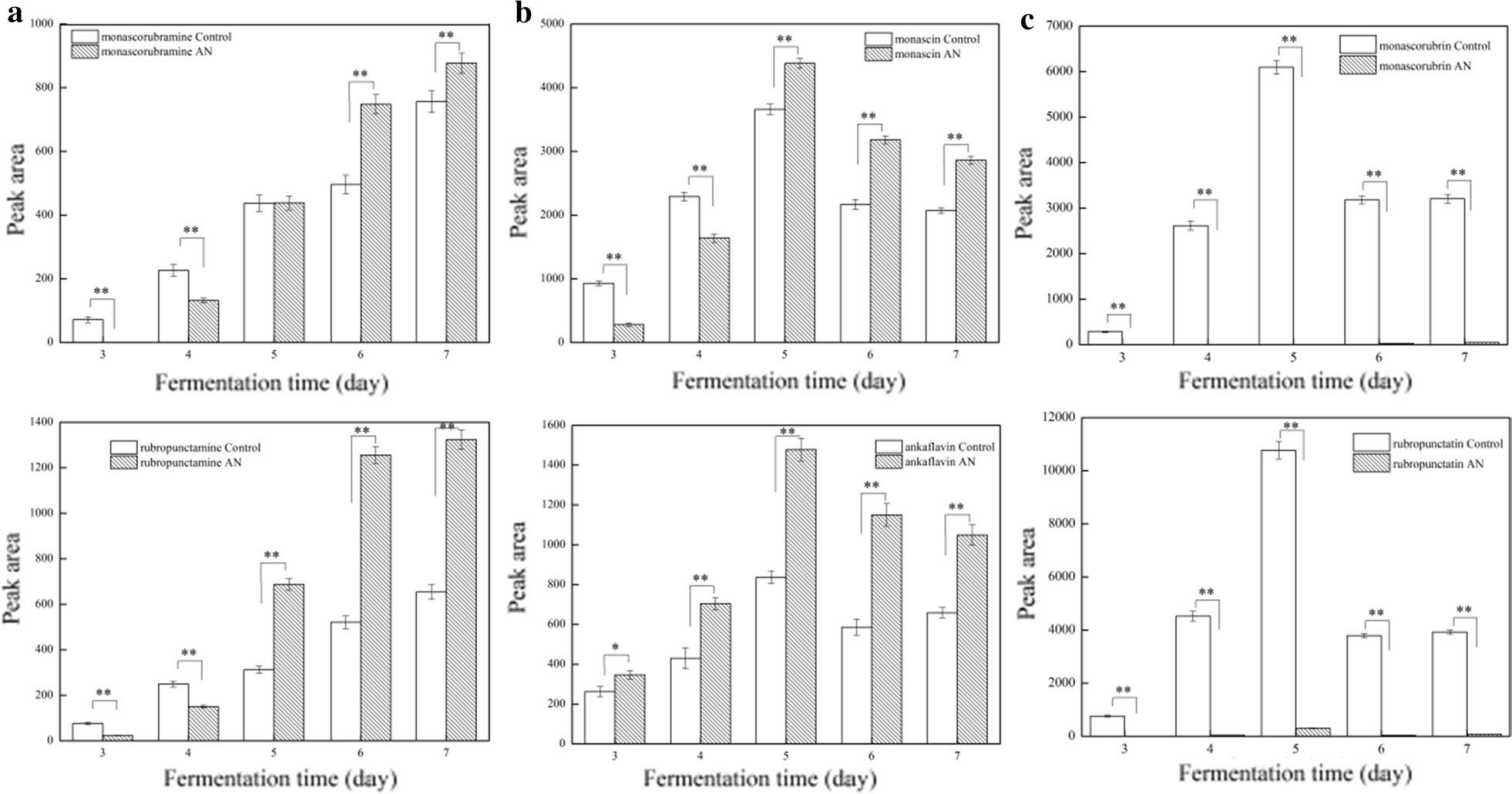
Effects of AN on the six classical MPs production. Yields of rubropunctatamine (R1) and monascorubramine (R2) (**a**), monascin (Y1) and ankaflavin (Y2) (**b**), rubropunctatin (O1) and monascorubrin (O2) (**c**) from the 3rd to 7th day of fermentation were assessed by relative peak areas provided by HPLC. The data were represented as the mean ± SD (n = 3). *P < 0.05, **P < 0.01 was compared with control

**Fig. 4:**
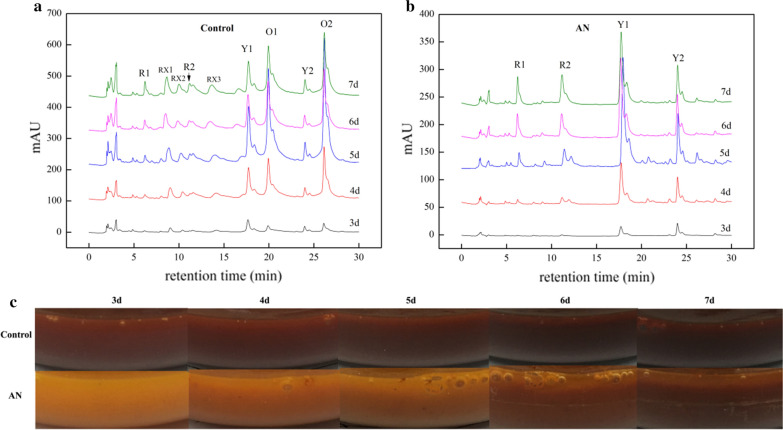
The HPLC profiles and images of fermentation broth. The profiles were corresponding to pigment extracts from mycelia of the control (**a**) and AN (**b**) treatment samples. The images (**c**) were photographed by fermentation broth from the 3rd to 7th day

The color characteristics of MPs are largely depended on the color and concentration of the components in the mixture. Therefore, the yields of six major compounds of MPs, yellows of monascin (Y1) and ankaflavin (Y2), oranges of monascorubrin (O1) and rubropunctatin (O2), and reds of monascorubramine (R1) and rubropunctamine (R2), were analyzed by HPLC, respectively, which provided an accurate determination of individual pigments. According to the peak areas, the yields of Y1, Y2 and O1, O2 were increased for the first 4 days, reaching the maximum amounts on the 5th day, and then decreased for the last 2 days (Fig. 3b, c). The corresponding absorbance data showed the similar trend of variation. The yields of R1 and R2 increased rapidly in the whole fermentation process (Fig. 3a). To be noted, when AN was added the amounts of O1 and O2 were greatly lowered to the undetectable level, whereas Y1, Y2 and R1, R2 production were evidently higher than the control sample from the 5th to the 7th day (Fig. 3). Interestingly, although O1 and O2 production were greatly inhibited by AN, the visual color of fermentation broth presented orange (Fig. 4c). Presumably, the mixture was consisted of red pigments overlapped with a certain amount of yellow pigments, contributing to the observed color.

There were obvious differences on the HPLC profiles between the control and AN samples. The peak of O1, O2 and a series of low-level amounts peaks, which emerged at the first 15 min of retention time, such as RX1, RX2 and RX3, but disappeared when subjected to AN. The spectrum recognized RX1, RX2 and RX3 as red pigments (Additional file 1: Fig. S1).

### AN regulated the expression of key genes involved in biosynthesis of MPs

**Fig. 5:**
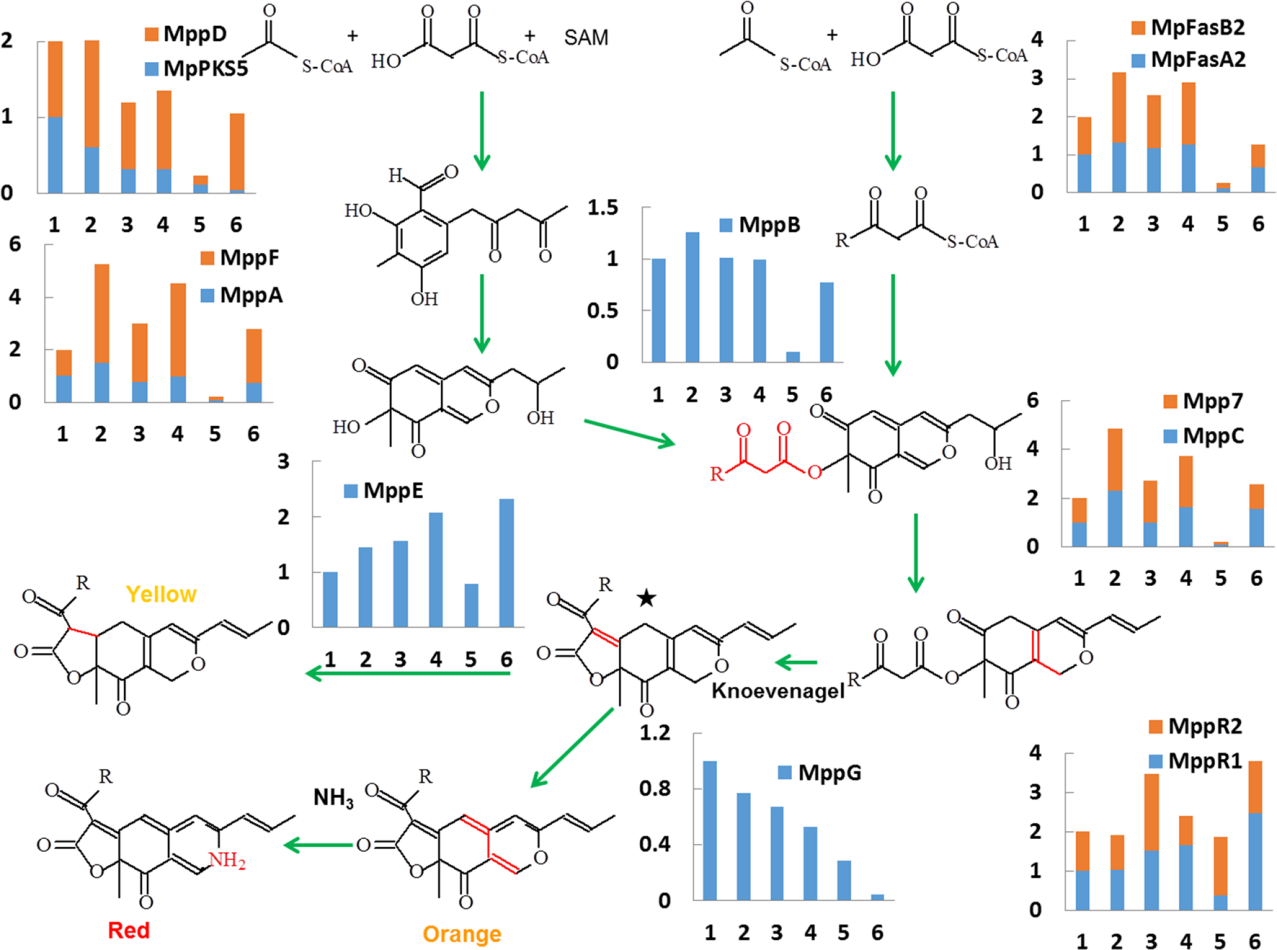
Expression levels of pigments biosynthetic genes in proposed biosynthetic pathway of MPs. The expression levels of 13 genes on the 3rd, 5th, and 7th day of fermentation of the control and AN treatment samples were analyzed by RT-qPCR. The control samples were 1 (3 days), 3 (5 days), 5 (7 days), and the AN samples were 2 (3 days), 4 (5 days), 6 (7 days). The transcriptional levels were normalized to those of the actin gene. The mRNA levels on the 3rd day of control were used as the reference value. The assay was repeated by three times

Up to now a systematic analysis of the MPs biosynthetic pathway had been provided (Chen et al. 2017). To investigate the effects of AN on MPs biosynthesis at the molecular level, we analyzed the expression level of 13 genes involved in major step for the classical yellow, orange and red pigment biosynthesis by RT-qPCR. The relative expression levels of *MpPKS5*, *MpFasA2*, *MpFasB2*, *mppA*, *mppB*, *mppC*, *mppD*, *mppG*, *mppE*, *mppF*, *mpp7, mppR1* and *mppR2* were monitored on the 3rd, 5th, and 7th days of fermentation (Fig. 5).

*MpPKS5*, polyketide synthase gene, is in charge of all *Monascus* azaphilone pigments related substances biosynthesis. The expression level of it with AN supplement was relatively lower than the control sample, which was positively correlated with the total MPs production according to absorbance (Balakrishnan et al. 2013). MppD, putative serine hydrolases, MppA, ketoreductase, as well as MppF, FAD-dependent monooxygenas are involved in polyketide chromophore production (Balakrishnan et al. 2015; Bijinu et al. 2014). In another branch, MpFasA2/MpFasB2, catalyzes the reaction of acetic acid with malonic acid to produced β-keto fatty acid (Balakrishnan et al. 2014b). MppB, acetyltransferase, transfers the fatty acyl chain to polyketide chromophore (Zabala et al. 2012).On the main pathway, Knoevenagel aldol condensations establish the tricyclic carbon skeletons that will serve as the common precursors for the classical yellow, orange and red pigments. Mpp7 and MppC (NAD(P)H-dependent oxidoreductase) assist in Knoevenagel aldol condensations (Balakrishnan et al. 2014a; Bijinu et al. 2014). Recent study showed that the deduced intermediates (yellow pigments; marked with asterisk in Fig. 5) might be the key branch point for the synthesis of the classical MPs (Chen et al. 2017). To sum up, these genes above-mentioned (MppA/B/C/D/F/7, MpFasA2/B2) are all involved in the precursors biosynthesis, of which the expression levels were all up-regulated with AN treatment. In term of MPs conversion, MppE, enoyl reductase, is related to form two classical yellow pigments by reduction of double bond, while MppG, FAD-dependent oxidoreductase, affords the orange pigments by restoring the double bond (Balakrishnan et al. 2017a, b, c). The expression levels of *mppE* and *mppD* performed with AN were higher and lower than the control sample, which were in accordance with the two yellow (Y1, Y2) and orange pigments (O1, O2) productions, respectively. MppR1 and MppR2, two transcription factors of azaphilone pigment gene cluster, were up-regulated and down-regulated compared to the control sample, respectively (Balakrishnan et al. 2013) (Fig. 5). It was demonstrated that AN led to the variation of the major components of MPs through regulating these genes expressions.

### AN increased the extracellular polysaccharide production

In the fermentation process we found the amounts of viscous substances appeared on the surface of mycelium. Accordingly, the extracellular polysaccharide production was investigated in this work (Fig. 1b). The yields of extracellular polysaccharide obtained with AN addition were significantly higher than the control sample, and reached to the maximum amount of 3.42 mg/mL on the 5th day of fermentation, which were approximately 5 times compared to the corresponding control sample. The results suggested that AN induced a large amount of extracellular polysaccharide biosynthesis.

## Discussion

The major MP products currently in China are appeared by red or deep red, such sausage, fermented bean curd, red yeast rice, etc. However, the fast-growing food industry, are in great need of various color characteristics of MPs. Many reports demonstrated that nitrogen, the pivotal nutrient factors, significantly influences MPs biosynthesis (Carels and Shepherd 1977; Chen and Johns 1993; Shi et al. 2015). In this study, the fermentation broth of *M. purpureus* M9 with AN supplement presented from light orange to orangish red during fermentation. We found that the reduction of MPs categories and conversion of the major components might be reponsible for the color characteristics variation.

MPs, the complex mixtures, have a package of components, which all contributes to the final color (Lv et al. 2017). The 6 classical components, 2 yellow (O1, O2), 2 red (R1, R2), and 2 orange pigments (O1, O2), plus a series of pigments with low-level amounts, were detected by HPLC after fermentation of M9 (Fig. 4a). In contrast, the peaks pattern resulted from AN treatment was obviously different from the control sample. A series of small peaks located on the first 15 min of eluted time were missing, which indicated the categories of MPs were reduced (Fig. 4b). Such result was in agreement with the absorbance data, which showed the concentration of MPs with AN supplement were significantly lowered. Particularly, the orange and red pigments were reduced by 67.1% and 82.3%, respectively (Fig. 2).

In addition, the peak areas of 2 orange pigments, O1 and O2, dropped down to the undetectable level when AN was added, whereas the peak areas of the yellow (Y1 and Y2) and the red (R1 and R2), were enhanced (Fig. 4b). According to the recent reports, the six classical MPs are originated from the same deduced intermediates. The 2 yellow pigments are formed by the reduction of double bonds, while the 2 orange pigments are produced by creating double bond. The amines directly react with the orange pigments to generate the red pigments by non-enzymatic reactions (Chen et al. 2017). Thus AN might trigger the transformations among yellow, orange and red pigments, resulting in the variation of pigmental contents.

To be noted, the expression levels of pigments biosynthetic genes further confirmed the above -mentioned hypothesis. The expression level of *mppG*, responsible for orange pigments biosynthesis, was significantly down-regulated with AN supplement. The metabolic flux might go towards the shunt pathway of yellow or red pigments. Therefore, the increased yellow pigments production is accompanied with the up-regulated *mppE* expression. Moreover, a large amount of NH_3_ units were obtained from AN-containing fermentation broth. Orange pigments are likely to react with amines, resulting in the increased red pigments yield. Thereby the expressions of pigment biosynthetic genes were regulated by AN, leading to the conversion of the major components of MPs.

In general, the effects of AN on color characteristic of MPs can be partially attributed to reduction of MPs categories and conversion of the major components. The fermentation broth of M9 with AN treatment exhibited the color from orange to orangish red in the time-dependent manner, whereas the control sample was appeared to be from red to deep red during fermentation (Fig. 4c). Neverthless, the color change of the intracellular extracts of M9 displayed the similar trends. According to the results from CIELAB colorimetric system, the hue angles of pigments with AN supplement were about 44–80 in the fermentation process, and the control samples were shown as 25–40, which represented orange and red, respectively (Table 1). UV–VIS spectrometry based on the characteristic absorbance of samples is commonly utilized for measuring the concentration of different pigments. However, due to the complexity of MP compositions, the solo spectrometric methodology could not distinguish each component with accuracy. Subsequently, in the present work, a much more efficient HPLC approach coupled with spectrometry was used to analyze individual MP components. Relative quantitation of each MP component was achieved by the measurement of HPLC peak areas. Hence, the preliminary mechanism of AN regulating color characteristics of MPs can be firstly disclosed in our data.

In order to further reveal the mechanistic basis for the changes of color characteristics, fermentation environmental factors were also investigated, such as biomass, pH value of fermentation broth, and extracellular polysaccharide yields. Especially, AN resulted in acidic condition in the whole fermentation process, in which the pH values of the fermentation broth were ranged from 3.0 to 5.0 (Fig. 1a). The acidic environment might be detrimental to partial secondary metabolites biosynthesis of *Monascus*, for the concentration of MPs and citrinin being decreased. In our previous study, it was found that no citrinin was detected by HPLC with a certain amount of AN supplement (data are not shown). Some reports showed that low pH inhibited citrinin production (Kang et al. 2014). Further, in the current study, the categories of MPs were reduced, including some low-content pigments and 2 classical orange ones. The yields of O1 and O2 reached to the undetectable level according to HPLC peak pattern (Fig. 4b). However, the productions of 2 yellow pigments (Y1, and Y2) were not affected. It’s worth noting that AN has the dual functions to alter the appearance of MPs from red to orange, as well as significantly inhibit the citrinin production. The polysaccharides were also the secondary metabolites of *Monascus* (Wang et al. 2014). The yields of extracellular polysaccharides with AN supplement were approximately 5 times more than that of the control samples, a fact suggests that the extracellular polysaccharide might serve as a protective agent for *Monascus* to resist acidic adversity. Moreover, biomass results showed that low pH almost do not influence the growth of *Monascus*.

In conclusion, AN regulated the color characteristics of MPs from red to orange in this work. The preliminary mechanisms were systematically investigated through a series of stepwise techniques. The change in color characteristics could be mainly attributed to the reduction of categories of pigments and the conversion of major components. The results in this study could provide useful data to produce MPs with the improved color characteristics.

## Supplementary information

**Additional file 1.** Tab. S1 Primers for RT-qPCR analyzing pigment biosynthetic genes. Fig. S1 The mass spectra and spectrum of Rubropunctatamine (R1) and Monascorubramine (R2), Monascin (Y1), and Ankaflavin (Y2), Rubropunctatin (O1) and Monascorubrin (O2), and three low-level amounts red pigments RX1, RX2, and RX3.

## Data Availability

We conducted experiments and data generated. All data is shown in figures, tables and additional data.

## References

[CR1] Balakrishnan B, Karki S, Chiu SH, Kim HJ, Suh JW, Nam B, Yoon YM, Chen CC, Kwon HJ (2013). Genetic localization and in vivo characterization of a *Monascus* azaphilone pigment biosynthetic gene cluster. Appl Microbiol Biotechnol.

[CR2] Balakrishnan B, Chen CC, Pan TM, Kwon HJ (2014). Mpp7 controls regioselective Knoevenagel condensation during the biosynthesis of *Monascus* azaphilone pigments. Tetrahedron Lett.

[CR3] Balakrishnan B, Kim HJ, Suh JW, Chen CC, Liu KH, Park SH, Kwon HJ (2014). *Monascus* azaphilone pigment biosynthesis employs a dedicated fatty acid synthase for short chain fatty acyl moieties. J Korean Soc for Appl Biol.

[CR4] Balakrishnan B, Chandran R, Park SH, Kwon HJ (2015). A new protein factor in the product formation of non-reducing fungal polyketide synthase with a C-terminus reductive domain. J Microbiol Biotechnol.

[CR5] Balakrishnan B, Lim YJ, Hwang SH, Lee DW, Park SH, Kwon HJ (2017). Selective production of red azaphilone pigments in a *Monascus purpureus mppDEG* deletion mutant. J Appl Biol Chem.

[CR6] Balakrishnan B, Park SH, Kwon HJ (2017). Inactivation of the oxidase gene *mppG* results in the selective loss of orange azaphilone pigments in *Monascus purpureus*. Appl Biol Chem.

[CR7] Balakrishnan B, Park SH, Kwon HJ (2017). A reductase gene *mppE* controls yellow component production in azaphilone polyketide pathway of *Monascus*. Biotechnol Lett.

[CR8] Bijinu B, Suh JW, Park SH, Kwon HJ (2014). Delineating *Monascus* azaphilone pigment biosynthesis: oxidoreductive modifications determine the ring cyclization pattern in azaphilone biosynthesis. RSC Adv.

[CR9] Calvo C, Salvador A (2002). Comparative study of the colorants *Monascus* and cochineal used in the preparation of gels made with various gelling agents. Food Hydrocoll.

[CR10] Carels M, Shepherd D (1977). The effect of different nitrogen sources on pigment production and sporulation of *Monascus* species in submerged, shaken culture. Can J Microbiol.

[CR11] Chen MH, Johns MR (1993). Effect of pH and nitrogen source on pigment production by *Monascus purpureus*. Appl Microbiol Biotechnol.

[CR12] Chen W, He Y, Zhou Y, Shao Y, Feng Y, Li M, Chen F (2015). Edible filamentous fungi from the species *Monascus*: early traditional fermentations, modern molecular biology, and future genomics. Compr Rev in Food Sci Food Saf.

[CR13] Chen D, Xue Y, Chen M, Li Z, Wang C (2016). Optimization of submerged fermentation medium for citrinin-free monascin production by Monascus. Prep Biochem Biotechnol.

[CR14] Chen W, Chen R, Liu Q, He Y, He K, Ding X, Kang L, Guo X, Xie N, Zhou Y (2017). Orange, red, yellow: biosynthesis of azaphilone pigments in *Monascus* fungi. Chem Sci.

[CR15] Cuesta G, Suarez N, Bessio MI, Ferreira F, Massaldi H (2003). Quantitative determination of pneumococcal capsular polysaccharide serotype 14 using a modification of phenol–sulfuric acid method. J Microbiol Methods.

[CR16] Dubois M, Gilles KA, Hamilton JK, Pt Rebers, Smith F (1956). Colorimetric method for determination of sugars and related substances. Anal Chem.

[CR17] Feng Y, Shao Y, Chen F (2012). *Monascus* pigments. Appl Microbiol Biotechnol.

[CR18] Hsu WH, Lee BH, Liao TH, Hsu YW, Pan TM (2012). *Monascus*-fermented metabolite monascin suppresses inflammation via PPAR-γ regulation and JNK inactivation in THP-1 monocytes. Food Chem Toxicol.

[CR19] Jung H, Kim C, Kim K, Shin CS (2003). Color characteristics of Monascus pigments derived by fermentation with various amino acids. J Agric Food Chem.

[CR20] Jůzlová P, Martinkova L, Křen V (1996). Secondary metabolites of the fungus *Monascus*: a review. J Ind Microbiol.

[CR21] Kang B, Zhang X, Wu Z, Wang Z, Park S (2014). Production of citrinin-free *Monascus* pigments by submerged culture at low pH. Enzyme Microb Technol.

[CR22] Kim C, Jung H, Kim YO, Shin CS (2006). Antimicrobial activities of amino acid derivatives of Monascus pigments. FEMS Microbiol Lett.

[CR23] Kim JH, Kim HJ, Kim C, Jung H, Kim YO, Ju JY, Shin CS (2007). Development of lipase inhibitors from various derivatives of monascus pigment produced by *Monascus* fermentation. Food Chem.

[CR24] Lin WY, Chang JY, Hish CH, Pan TM (2007). Profiling the *Monascus pilosus* proteome during nitrogen limitation. J Agric Food Chem.

[CR25] Lin CH, Lin TH, Pan TM (2017). Alleviation of metabolic syndrome by monascin and ankaflavin: the perspective of *Monascus* functional foods. Food Funct.

[CR26] Luo F, Cheng SC, Cai JH, Wei BD, Zhou X, Zhou Q, Zhao YB, Ji SJ (2019). Chlorophyll degradation and carotenoid biosynthetic pathways: gene expression and pigment content in broccoli during yellowing. Food Chem.

[CR27] Lv J, Zhang BB, Liu XD, Zhang C, Chen L, Xu GR, Cheung PCK (2017). Enhanced production of natural yellow pigments from *Monascus purpureus* by liquid culture: the relationship between fermentation conditions and mycelial morphology. J Biosci Bioeng.

[CR28] Patakova P (2013). *Monascus* secondary metabolites: production and biological activity. J Ind Microbiol Biotechnol.

[CR29] Peters B, Smuła-Ostaszewska J (2012). Simultaneous prediction of potassium chloride and sulphur dioxide emissions during combustion of switchgrass. Fuel.

[CR30] Sen T, Barrow CJ, Deshmukh SK (2019). Microbial pigments in the food industry—challenges and the way forward. Front Nutr.

[CR31] Shi K, Song D, Chen G, Pistolozzi M, Wu Z, Quan L (2015). Controlling composition and color characteristics of *Monascus* pigments by pH and nitrogen sources in submerged fermentation. J Biosci Bioeng.

[CR32] Su NW, Lin YL, Lee MH, Ho CY (2005). Ankaflavin from *Monascus*-fermented red rice exhibits selective cytotoxic effect and induces cell death on Hep G2 cells. J Agric Food Chem.

[CR33] Upadhyaya L, Singh J, Agarwal V, Pandey A, Verma SP, Das P, Tewari R (2015). Efficient water soluble nanostructured ZnO grafted O-carboxymethyl chitosan/curcumin-nanocomposite for cancer therapy. Process Biochem.

[CR34] Velmurugan P, Chae JC, Lakshmanaperumalsamy P, Yun BS, Lee KJ, Oh BT (2009). Assessment of the dyeing properties of pigments from five fungi and anti-bacterial activity of dyed cotton fabric and leather. Color Technol.

[CR35] Wang P, Chen D, Jiang D, Dong X, Chen P, Lin Y (2014). Alkali extraction and in vitro antioxidant activity of *Monascus* mycelium polysaccharides. J Food Sci Technol.

[CR36] Yang Y, Liu B, Du X, Li P, Liang B, Cheng X, Du L, Huang D, Wang L, Wang S (2015). Complete genome sequence and transcriptomics analyses reveal pigment biosynthesis and regulatory mechanisms in an industrial strain, *Monascus purpureus* YY-1. Sci Rep.

[CR37] Zabala AO, Xu W, Chooi YH, Tang Y (2012). Characterization of a silent azaphilone gene cluster from *Aspergillus niger* ATCC 1015 reveals a hydroxylation-mediated pyran-ring formation. Chem Biol.

