# Supplementary material for: Ammonium nitrate regulated the color characteristic changes of pigments in Monascus purpureus M9
Source: AMB Express. 2021 Jan 4;11:3. doi: 10.1186/s13568-020-01165-6 (PMC7782668; doi:10.1186/s13568-020-01165-6)
Supplement: Supplementary file 1 — Additional file 1. Tab. S1 Primers for RT-qPCR analyzing pigment biosynthetic genes. Fig. S1 The mass spectra and spectrum of Rubropunctatamine (R1) and Monascorubramine (R2), Monascin (Y1), and Ankaflavin (Y2), Rubropunctatin (O1) and Monascorubrin (O2), and three low-level amounts red pigments RX1, RX2, and RX3. [file 13568_2020_1165_MOESM1_ESM.docx]

***AMB Express***

Ammonium nitrate regulated the color characteristic changes of pigments in *Monascus purpureus* M9

**Di Chen^1*^, Yurong Wang^2^, Mianhua Chen^2^, Pei Fan^1^, Guiling Li^1^, Changlu Wang^2*^**

^1^ College of Biological Engineering, Henan University of Technology, No.100, Lianhua Street, High-tech Industrial Development Area, Zhengzhou, 450001, P. R. China.

^2^  Key Laboratory of Food Nutrition and Safety, Ministry of Education, College of Food Engineering and Biotechnology, Tianjin University of Science and Technology, No. 29, 13th Avenue, TEDA, Tianjin, 300457, P. R. China.

**^*^Corresponding author**

Di Chen

E-mail: [chendi.1126@163.com](mailto:chendi.1126@163.com)

Changlu Wang

E-mail: [clw123@tust.edu.cn](mailto:clw123@tust.edu.cn)

Table S1. Primers for RT-qPCR analyzing pigment biosynthetic genes

| primers | sequence |
| --- | --- |
| *β-actin*F | 5'-TTCGAGACCTTCAACGCCC-3' |
| *β-actin*R | 5'-ACCCTCGTAGATGGGAACGA-3' |
| *MpPKS5rt*F | 5'-TGTCCGACGAGTTTCTGCAA-3'  GACCCAGATCCCACCTACCT  -3’ |
| *MpPKS5rt*R | 5'-TATCAACGCTGCTTGGGCAT-3' |
| *mppR1rt*F | 5'-TCTGCAGTATGCCATGTGGG-3' |
| *mppR1rt*R | 5'-ATGGCACCGTCACTTAGCTC-3' |
| *mppArt*F | 5'-GGTCAATCTTCGTGGACCGT-3' |
| *mppArt*R | 5'-AGTCGGGTTGAGAAGATGCG-3' |
| *mppBrt*F | 5'-CGTCTCGCCCGATAACTTCA-3' |
| *mppBrt*R | 5'-TTGACAGACGGGTCGAAGTC-3' |
| *mppCrt*F | 5'-CAGTCCTCGTCCCTTCCAGT-3' |
| *mppCrt*R | 5'-CCACGGTGAAGGATGTCGAG-3' |
| *mppGrt*F | 5’- TCAACACGGGAGATGCTGTC-3' |
| *mppGrt*R | 5'-GCCAAAGGACAGGAGCAGAT-3' |
| *mppDrt*F | 5'-AGGAGTTCGGGCCATTCAAG-3' |
| *mppDrt*R | 5'-TCTTCGTCCATGGCATCTCG-3' |
| *mppErt*F | 5'-CTTCCCGATGCCGTTGTGAT-3' |
| *mppErt*R | 5'-CGTCTCGTGGATCATCTCGT-3' |
| *mppR2rt*F | 5'-ACGAAACCCTCCATGACACC-3' |
| *mppR2rt*R | 5'-TGCAGACAGCCTTGTGGTAG-3' |
| *FasA2rt*F | 5'-ATGGATCGCCCGATCTTGTC-3' |
| *FasA2rt*R | 5'-CTTTGTCGAGTCCGCTGGAT-3' |
| *FasB2rt*F | 5'-CCTCCAGGGATTACAACCCG-3' |
| *FasB2rt*R | 5'-ATTCAATGCCAGGTGCTCCA-3' |
| *mppFrt*F | 5'-TAGCATCGTACGTCGCCAAC-3' |
| *mppFrt*R | 5'-TGTCCTGGCAGCTGTATTGG-3' |
| *Mpp7rt*F | 5'-GCCCATCTTCAAGTACCAC-3' |
| *Mpp7rt*R | 5'-CCTTCAAGCCATTCTCATAG-3' |


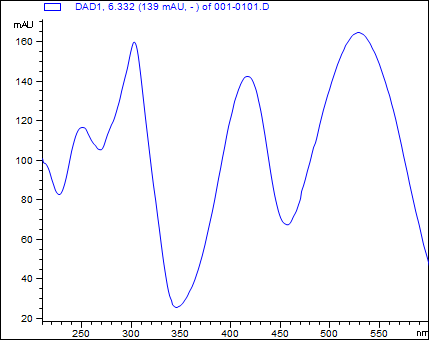

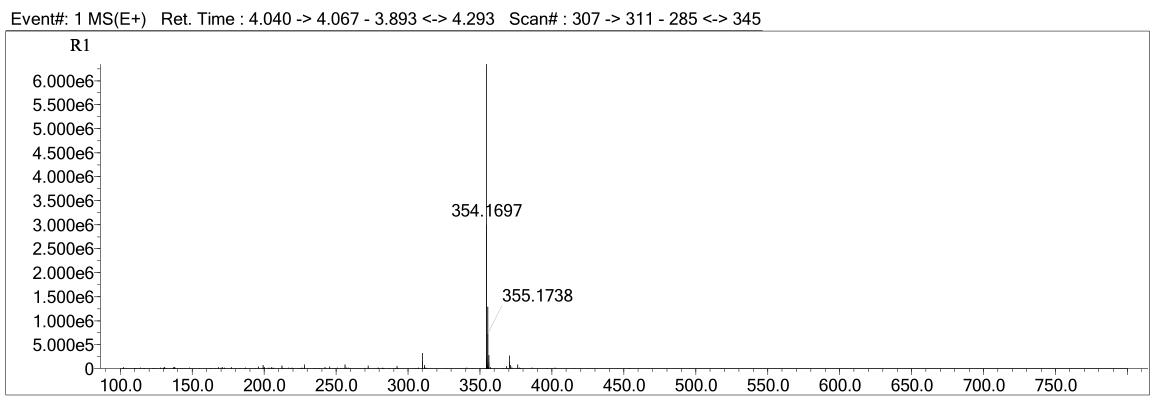


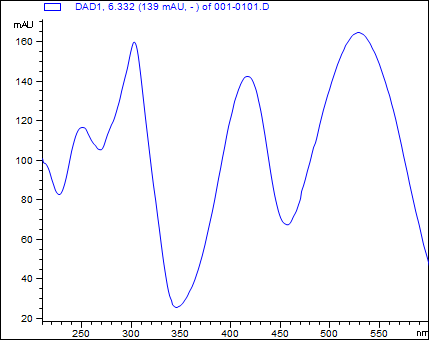

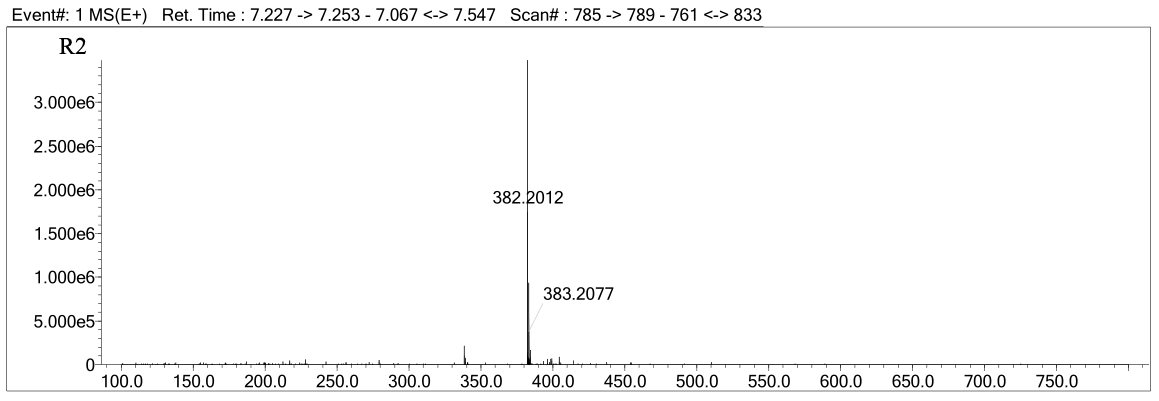


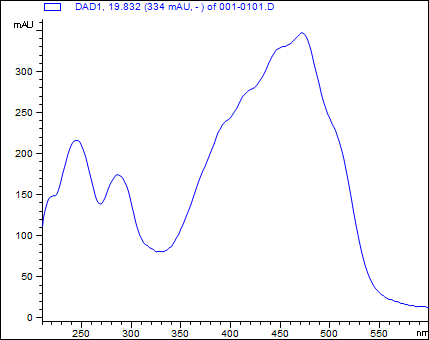

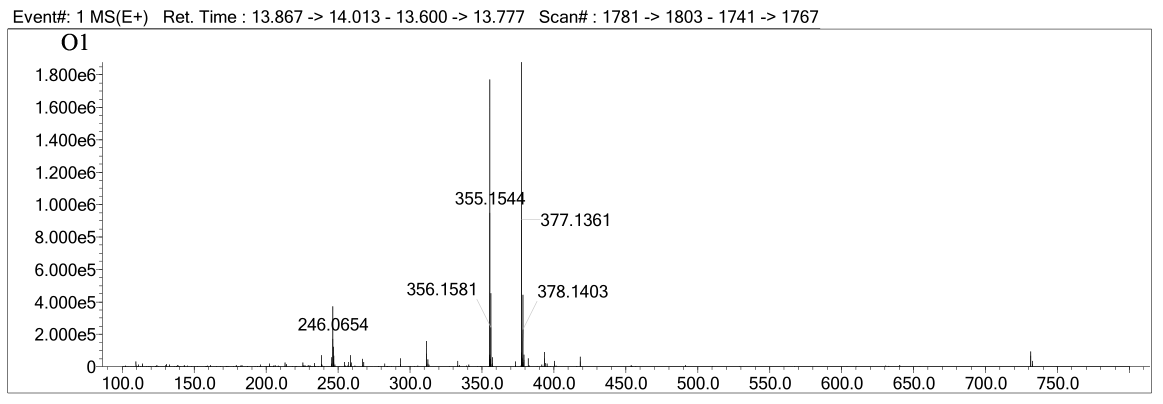


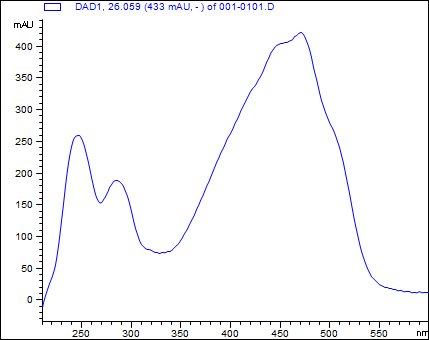

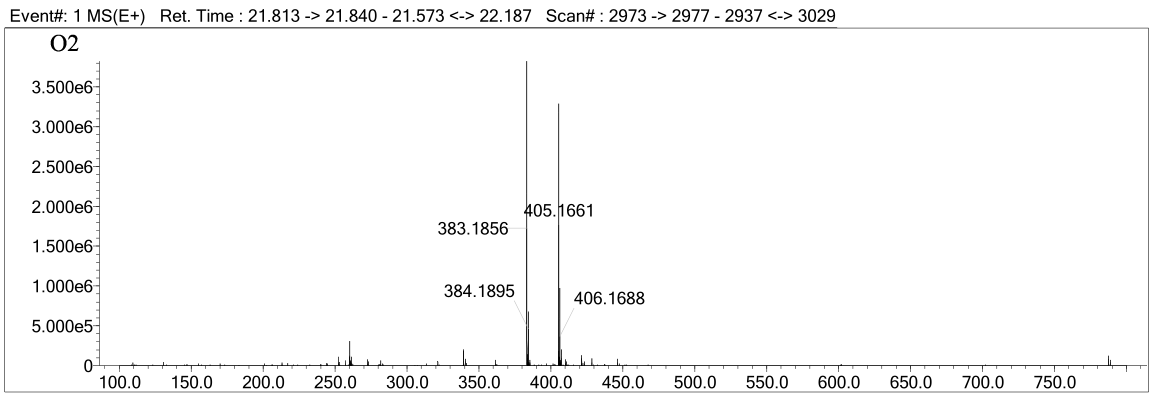


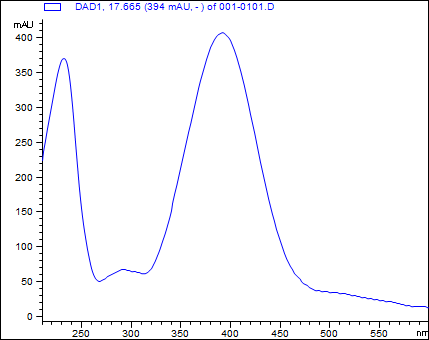

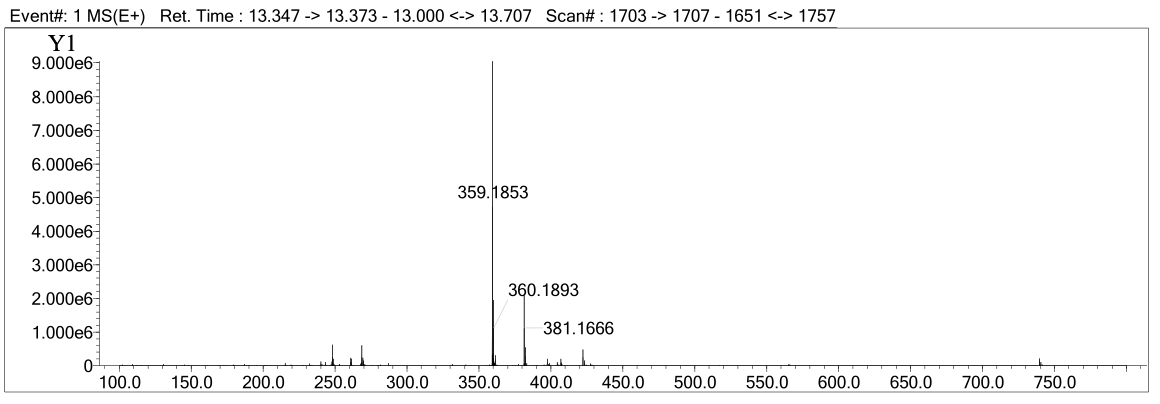


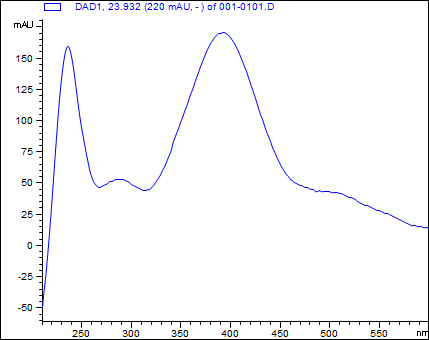

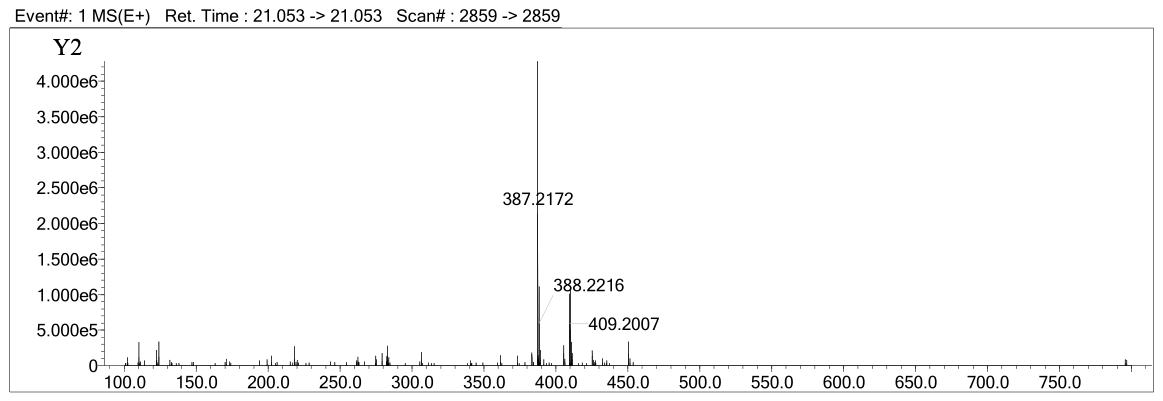


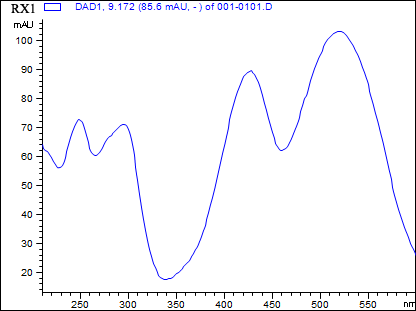

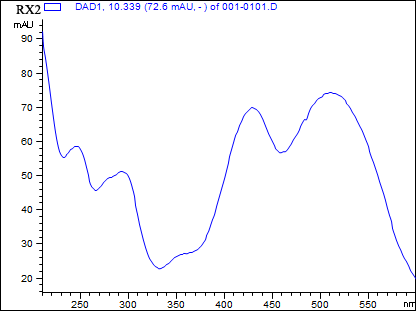

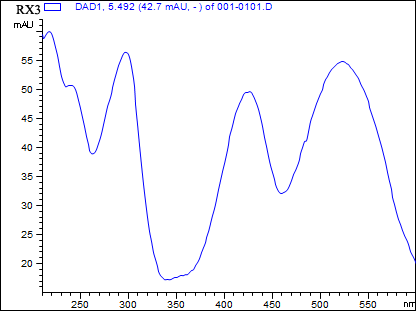


Figure. S1 The mass spectra and spectrum of Rubropunctatamine (R1) and Monascorubramine (R2), Monascin (Y1), and Ankaflavin (Y2), Rubropunctatin (O1) and Monascorubrin (O2), and three low-level amounts red pigments RX1, RX2, and RX3.
